# Supplementary material for: Metabolism of Reactive Oxygen Species in Osteosarcoma and Potential Treatment Applications
Source: Cells. 2019 Dec 30;9(1):87. doi: 10.3390/cells9010087 (PMC7017125; doi:10.3390/cells9010087)
Supplement: Supplementary file 1 [file cells-09-00087-s001.pdf]

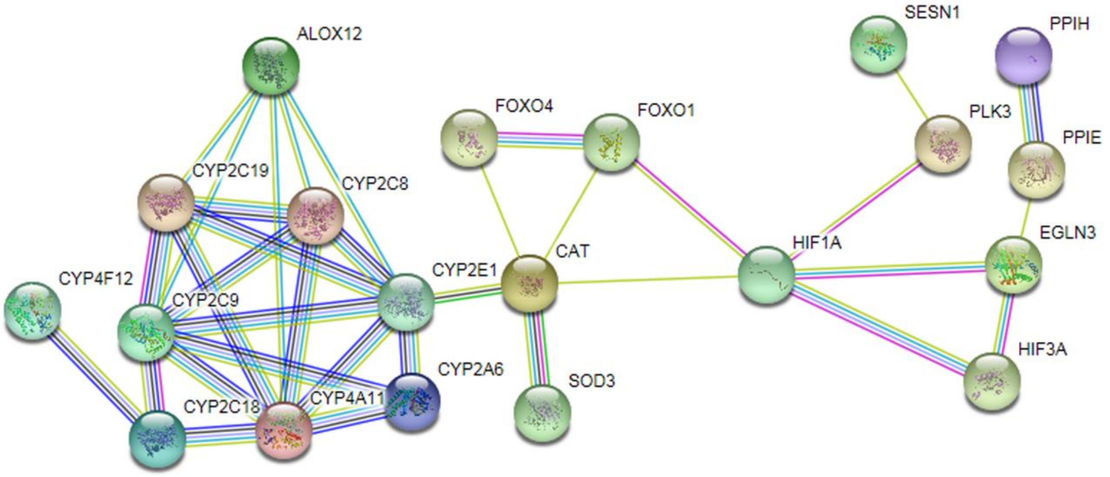

Supplemental Figure S1. Protein-protein association network of the ROS metabolism regulation pathway in a STRING analysis. HIF-1α was selected as the input.

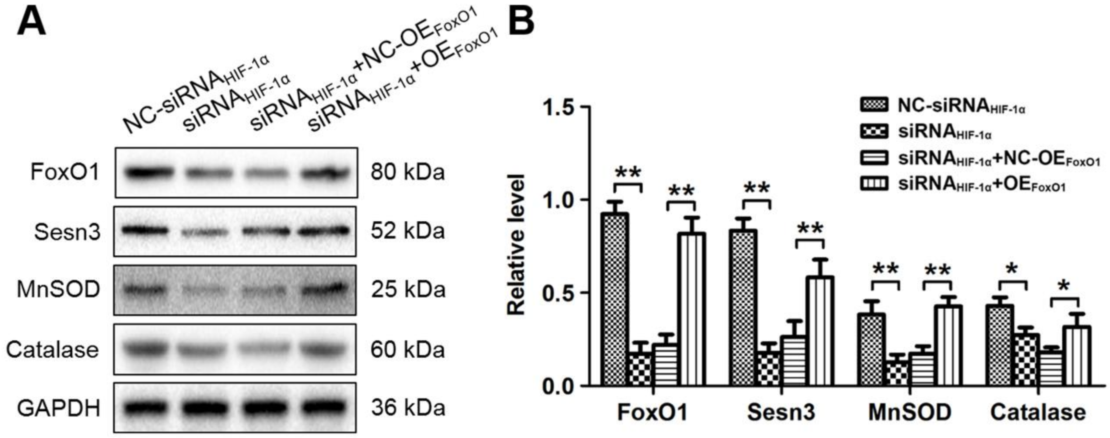

Supplemental Figure S2. FoxO1, MnSOD, catalase and Sesn3 expression was rescued by FoxO1 overexpression in HIF-1α siRNA cell lines. (**A**) Western blot analyses were performed to evaluate = FoxO1, MnSOD, catalase, and Sesn3 protein expression in MG63 cells with FoxO1 overexpression. (**B**) Quantitative analysis of the protein levels. * $p < 0.05$, ** $p < 0.01$.

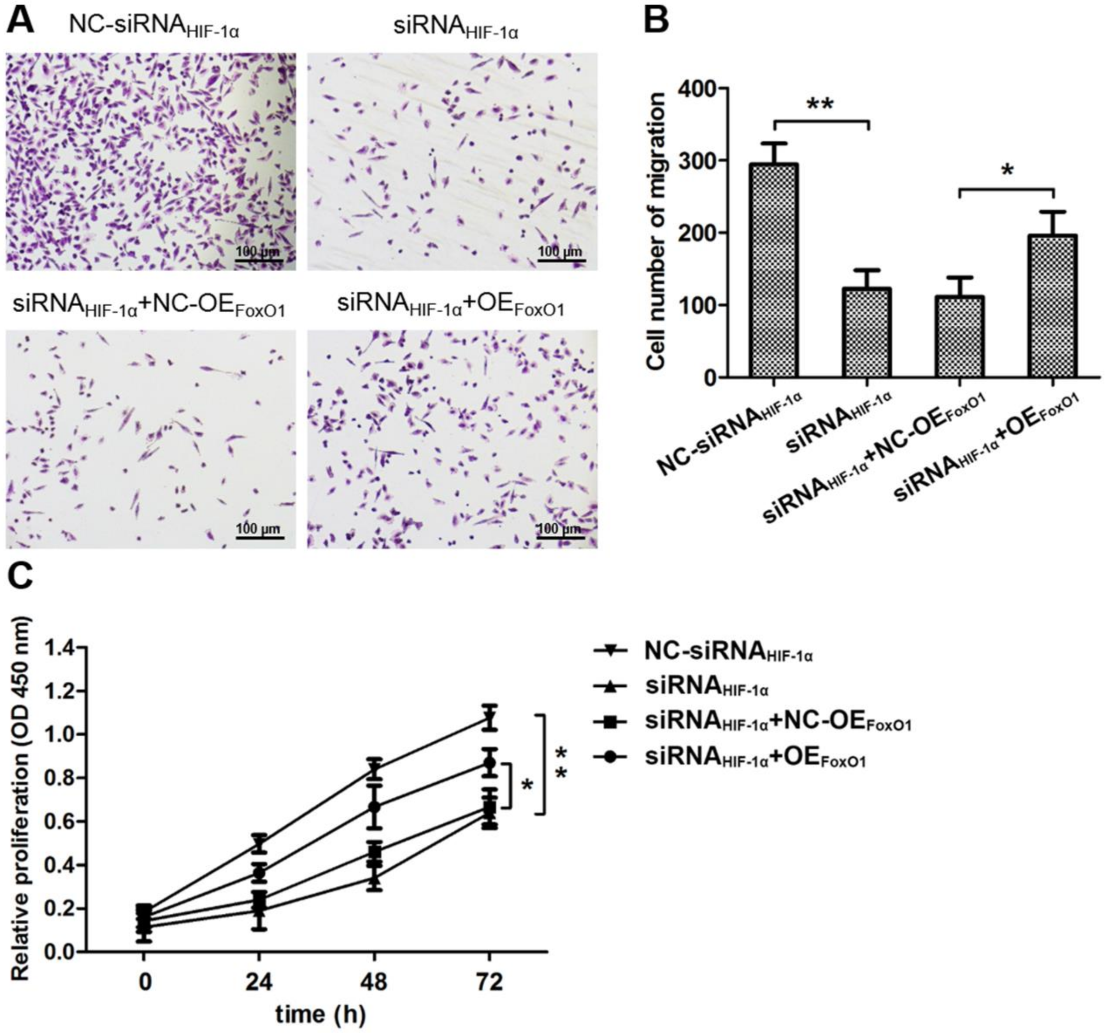

Supplemental Figure S3. FoxO1 overexpression rescued the migration and proliferation of HIF-1*α* siRNA cell lines. (**A**) and (**B**) Transwell analyses of the migrated HIF-1*α* siRNA cells and FoxO1 overexpression cells. The cells were allowed to migrate for 12 h. (**C**) A CCK-8 assay was performed to monitor the proliferation level of HIF-1*α* siRNA cells and FoxO1 overexpression cells at 0, 24, 48, 72 h. (* $p < 0.05$; ** $p < 0.01$).
